# The “Man of the Fork”

**Published:** 1874-12

**Authors:** 


The "Man of the Fork."—The young man who swallowed a fork last winter, in Paris, for a wager, and who is known there as the "homme a la fourchette," is, according to latest accounts, doing as well as can be expected, and suffers no inconvenience from the presence of the fork in his stomach, except after a full meal, when he is obliged to bend himself a little to the left side, which position affords him relief. His health not being compromised in any way, M. Labbe, the surgeon who attends him, has resolved upon abstaining from any operative interference, at least for the present.

